# Expression of 32/67-kDa laminin receptor in laminin adhesion-selected human colon cancer cell lines.

**DOI:** 10.1038/bjc.1998.3

**Published:** 1998

**Authors:** W. H. Kim, B. L. Lee, S. H. Jun, S. Y. Song, H. K. Kleinman

**Affiliations:** Department of Pathology, Seoul National University College of Medicine and Cancer Research Center, Korea.

## Abstract

**Images:**


British Journal of Cancer (1998) 77(1), 15–20
© 1998 Cancer Research Campaign

### Expression of 32/67-kDa laminin receptor in laminin adhesion-selected human colon cancer cell lines

**WH Kim[1], BL Lee[2], SH Jun[3], SY Song[4] and HK Kleinman[4]**

Departments of [1]Pathology and [2]Anatomy, Seoul National University College of Medicine and Cancer Research Center, Seoul 110-799, Korea; [3]Department of Surgery, Kyoungpook National University College of Medicine, Taegu 700-422, Korea; [4]Cell Biology Section, National Institute of Dental Research, National Institutes of Health, Bethesda, MD 20892, USA

**Summary** Laminin promotes the malignant phenotype, and the expression of certain laminin receptors is increased in malignancy. Previously, we demonstrated that a laminin-adhesive subclone of a human colon cancer cell line showed increased tumorigenicity in nude mice and increased affinity of the $\beta_1$ integrin for laminin relative to the laminin-non-adhesive subclone. The total amount of either $\beta_1$ integrin protein or mRNA did not increase. As levels of the 32/67-kDa laminin receptor (67LR) correlate with malignancy, we examined 67LR expression in the laminin adhesion-selected human colon cancer cells. The laminin-adhesive subclone, which was more tumorigenic in both heterotopic and orthotopic locations than in a laminin-non-adhesive subclone, showed cell-surface membrane staining of 67LR, whereas the laminin-non-adhesive subclone showed cytoplasmic staining of 67LR. No difference in either the amount of 67LR mRNA or the amount of protein was observed in the parental cells than in the laminin-adhesive and non-adhesive subclones. When assayed on a laminin affinity column, more 67LR molecules bound to the column with cell extracts from the laminin-adhesive subclone than was observed with the non-adhesive subclone. These findings suggest that the increased tumorigenicity of laminin adhesion-selected tumour cells might be due to an alteration in the distribution and/or adhesiveness of multiple receptors including 67LR and $\beta_1$ integrin.

**Keywords:** laminin; 32/67-kDa laminin receptor; laminin adhesion selection; colon cancer cell line; immunohistochemistry; affinity chromatography

Laminin, a major component of basement membrane, exhibits diverse biological activities in both normal cells and tumour cells (Kleinman et al, 1993). Co-injection of laminin and tumour cells increased tumorigenicity as well as metastasis (Terranova et al, 1984), whereas certain fragments decrease tumorigenicity. Adhesion of cancer cells to laminin is mediated through various cell-surface receptors, which may serve different functions. Among the known surface-binding proteins, the 32/67-kDa laminin receptor (67LR) was the first protein identified as a high-affinity receptor (Malinoff and Wicha, 1983). The expression of this 67LR is increased in several human cancers: 67LR is higher in colon cancer tissue than in adjacent normal tissue, both at the protein level and at the mRNA level (Horan-Hand et al, 1985; Cioce et al, 1991). The expression of this receptor correlates positively with tumour progression or Dukes' stage of colon cancer (Mafune et al, 1990). The molecular weight of this receptor is variable when examined with immunoreactive antibody by Western blot and molecular weights of 32, 38, 45, 54 and 67 kDa are observed. In contrast, only one mRNA at 1.1 kb has been detected. The reason for the different sizes is probably post-translational modifications but that has not yet been described.

Aznavoorian et al (1990) selected colon cancer cell lines according to their invasiveness through amnion. The isolated, highly invasive human colon cancer cell line migrated more strongly towards laminin in vitro and showed strong laminin-binding activity. Similarly, Yamamura et al (1993) selected malignant melanoma cells based on their adhesiveness to the YIGSR peptide, one of the biologically active sites of laminin. YIGSR-adherent melanoma cells were more malignant in nude mice than YIGSR-non-adherent cells. The molecular mechanisms underlying the biological difference have not been defined in the above two selected cell lines.

We selected a colon cancer cell line based on its adhesiveness to laminin (Jun et al, 1994). We have characterized the adherent and non-adherent cells in terms of their biological tumorigenic activity and $\beta_1$ integrin expression (Kim et al, 1995). The laminin-adhesive subclone was more tumorigenic and invasive, and showed membranous distribution of $\beta_1$ integrin. The $\beta_1$ integrin of the laminin-adhesive subclone had higher affinity to laminin than that of the laminin-non-adhesive subclone.

In this paper, the laminin adhesion-selected colon cancer cells were analysed for expression of 67LR. The results showed that differences in tumorigenicity might be caused by alterations in multiple laminin receptors, including $\beta_1$ integrin and 67LR.

### MATERIALS AND METHODS

### Cell culture and selection

Laminin and Matrigel were prepared from the Engelbreth–Holm–Swarm (EHS) tumour, which is known to produce a large amount of basement membrane. The preparation methods have been described elsewhere (Kleinman et al, 1986; Martin and Timpl, 1987). Antibodies to the 67LR were prepared against

Received 5 February 1997
Revised 10 June 1997
Accepted 11 June 1997

Correspondence to: WH Kim, Department of Pathology, Seoul National University College of Medicine, 28 Yongon-dong, Chongno-gu, Seoul 110-799, Korea either a synthetic peptide from the deduced sequence of amino terminus (HK-149) or a fusion protein from the carboxy terminus (HK-48) (Clement et al, 1990). Antibody against 110 kDa laminin receptor was prepared using purified protein from fetal mouse brain (Kleinman et al, 1991).

The establishment of ET human colon cancer cell line (also named LCC-C1) and selection of subclones has been described previously (Jun et al, 1994). In brief, a biopsy specimen from a colon adenocarcinoma (Dukes' stage B2) was minced, mixed with 0.5 ml of Matrigel (14 mg $ml^{-1}$) and injected subcutaneously into a nude mouse. The resulting tumour was harvested and again injected into another nude mouse after mincing and mixing with Matrigel. The tumour from the third xenograft was enzymatically digested with trypsin–EDTA (0.05% trypsin in 0.53 mM EDTA) to yield a single-cell suspension. These cells were cultured in RPMI-1640 growth medium (Gibco-BRL) containing 10% fetal calf serum (Hyclone), insulin (5 μg $ml^{-1}$), transferrin (5 μg $ml^{-1}$), selenium (5 μg $ml^{-1}$) penicillin (100 μg $ml^{-1}$), streptomycin (100 μg $ml^{-1}$) and gentamicin (50 μg $ml^{-1}$).

The selection procedure was as follows. The cells were detached from the plastic dish and dispersed with trypsin–EDTA; after washing to remove the trypsin–EDTA, the cells were plated onto a Nunc culture plate (150 mm diameter) which had been coated with 0.7 mg $ml^{-1}$ laminin solution for 1 h at 37°C. After a 1-h incubation on the laminin-coated plates, unattached cells were collected, cultured until they became confluent and harvested again. The selection procedures were repeated 26 times to yield the $ET^-$ cells. Similarly, the attached cells on the laminin-coated culture plates were repeatedly selected 35 times to yield $ET^+$ cells.

### Heterotransplantation

Parental ET cells, $ET^+$ cells or $ET^-$ cells were injected into the caecal wall of nude mice, with six mice used for each cell type. The cells at 70% confluency were enzymatically dispersed into single cells. Fifty thousand cells were suspended in 0.2 ml of Matrigel at 4°C. The mice were anaesthetized and the Matrigel–cell mixtures were injected into the wall of the caecum. The mice were sacrificed 30 days later and the histology of the caecal tumours was examined after haematoxylin–eosin staining.

### Attachment assay

Cell attachment was assessed in round-bottom 96-well culture plates. The plates were coated with varying amounts of laminin in 50 μl of Milli-Q water (Kim et al, 1994). After drying the laminin, the wells were blocked by the addition of 0.2 ml of 3% bovine serum albumin (BSA) in RPMI-1640 medium for 1 h and then washed with the 0.1% BSA solution. The cells, detached by trypsin–EDTA, were resuspended in 0.1% BSA in RPMI-1640 medium, added to the wells (40 000 cells in 0.2 ml) and incubated in a carbon dioxide incubator for 1 h. After incubation, the unattached cells were removed by inverting the plates and gentle tapping, and the attached cells were stained with 0.2% crystal violet solution in 20% methanol for 10 min. The optical density at 560 nm was measured after dissolving the stained cells with a 1% sodium dodecyl sulphate (SDS) solution.

### Western blot

Near-confluent cells were dissolved in 50 mM Tris-HCl buffer containing 150 mM sodium chloride, 1% Triton-X 100, 0.1% deoxycholic acid, 0.1% SDS and 2 mM phenylmethylsulphonyl fluoride (PMSF). Protein content was measured by the BCA method, and 50 μg of total cell lysate protein was separated on 7.5% SDS–polycrylamide gels under reducing conditions with 0.1 M DTT. The proteins were electroblotted to polyvinylidene difluoride paper and the blot was immersed in 50 mM Tris-HCl (pH 7.4) containing 150 mM sodium chloride, 0.1% Tween-20 and 5% non-fat dry milk for 1 h. The primary antibodies against 67LR (HK-149 or HK-48) (Clement et al, 1990) were diluted to 1:5000 and incubated for 2 h.

**Figure 1** Attachment of subclones to laminin-coated plates. The 96-well plates were coated with various amounts of laminin and attached cells were compared after staining with crystal violet

**Figure 2** Western blot of 67LR with HK-149 antibody. Cell lysates were electrophoresed on a 7.5% SDS gel, transferred to a nitrocellulose membrane and reacted with the HK-149 antibody. The three cell lines showed similar intensity of immunoreactivity, including the major 45-kDa band

© Cancer Research Campaign 1998

**Figure 3** Northern blot of mRNA from colon cancer cell lines with cDNA for 67LR. There was no difference of the amount of transcript between the three lanes

Biotinylated anti-rabbit IgG and avidine–biotin–peroxidase complex were subsequently added, and ECL (Amersham) was used to detect the immune complexes by chemiluminescence.

### Northern blot

Total RNA was extracted from near confluent cells. The cells were scraped into a solution of guanidine thiocyanate and RNA was purified by ultracentrifugation on a caesium chloride cushion. An aliquot (10 μg) of total RNA was run on a 1% agarose–formaldehyde gel and transferred to a nytran filter. A full length 32/67-kDa cDNA (Segui-Real et al, 1989), cloned from a human placental library, was obtained from Dr Y Yamada (NIH, MD, USA). The insert of 1.1 kb was [$^{32}$P]dCTP by random oligonucleotide primer extension. After hybridization and stringent washing, the filter was autoradiographed. Each blot was repeated twice with a different preparation of RNA.

### Immunohistochemistry

The cells were cultured on 13 mm round Thermanox cover slips (Nunc, Naperville, IL, USA) for 3 days and fixed with 4% formaldehyde. After washing, the cells were blocked with non-immune serum, then incubated with 67LR antibodies (either HK-48 or HK-149 at 1:800), followed by incubation with biotinylated anti-rabbit IgG. After washing, bound antibody was detected using an ABC immunostaining kit (Vector, Burlingame, CA, USA) and diaminobenzidine–hydrogen peroxide.

### Laminin affinity chromatography

Near-confluent colon cancer cells were homogenized in 100 mM Tris-HCl (pH 7.4) buffer containing 25 mM *n*-octyl-β-D-glucopyranoside, 150 mM sodium chloride, 2 mM PMSF, 1 mM manganese chloride and centrifuged at 10 000*g* for 15 min. The supernatant was incubated overnight at 4°C with laminin–Sepharose beads. The beads were then packed in a column and washed with the same buffer until protein elution stopped. The bound fractions were eluted with the same buffer containing 20 mM EDTA instead of manganese chloride. The eluates were dialysed, lyophilized and separated using 7.5% SDS–PAGE under reducing conditions. After electroblotting, western blot staining was performed with the HK-149 antibody. In addition, western blot with antibody against the 110-kDa laminin receptor (Kleinman et al, 1991) was performed.

**Figure 4** (**A**) Immunohistochemical staining of parenatal cell line with 67LR antibody. (**B**) Staining the ET(+) cells with the same antibody exhibited membraneous distrubution of 67LR. (**C**) ET(−) cells expressed the 67LR in the cytoplasm

### RESULTS

The attachment of the laminin-adherent, laminin-non-adherent and parental subclones was compared (Figure 1). The ET$^+$ subclone showed the strongest attachment to laminin at concentrations higher than 1 μg per well in the 96-well plate. ET$^-$ cells showed the least attachment among the three cells, although the difference between parental and ET$^-$ was not significant at concentrations higher than 1 μg per well.

In vitro morphology of ET$^+$ cells showed flattening of the cytoplasm around cell aggregates and formation of a central volcano-like structure as previously reported (Jun et al, 1994). The cell-to-cell border was conspicuous in the ET$^+$ cells. The ET$^-$ cells showed dome-shaped cell aggregates without flattening at the periphery and the volcano-like structure was not observed. The parental cells showed dome-shaped aggregates with inconspicuous cell borders.

© Cancer Research Campaign 1998 

**Figure 5** Western blot for the 67LR following laminin affinity chromatography. (**A**) The laminin-binding fractions were separated by SDS-PAGE followed by electroblotting and immunostaining with 67LR antibody. Several bands (arrows) including 67, 45 and 32 kDa are observed. (**B**) The same blot was stripped and immunostained with 110 kDa laminin receptor antibody. A similar level of expression was noted (arrowhead) between the two subclones

We compared the tumorigenicity of these tumour cells in the caecum of the nude mice. Orthotopic injection of tumour cells represents the best approach for measuring the invasiveness or metastatic potential of tumour cells (Togo et al, 1995). $ET^+$ tumour cells produced large caecal masses in all six mice, whereas parental cells developed tumours in three out of six mice and $ET^-$ cells produced tumours in two out of six mice. The tumour volume for the $ET^+$ cells was much greater (112.4 ± 40.9 $mm^3$) than that of either the parental cells (7.3 ± 4.2 $mm^3$) or the $ET^-$ cells (3.6 ± 1.9 $mm^3$). Histologically, the $ET^+$ cell-derived tumours were less differentiated and formed smaller glands with minute lumina. Tumours from $ET^-$ or parental cells were well-differentiated adenocarcinomas. The outer and inner diameter of the individual gland were larger than those of $ET^+$ tumours, and nuclei were elongated and slender. Tumours from $ET^+$ cells were moderately differentiated adenocarcinomas and the nuclei were ovoid. None of the caecally grown tumours metastasized to the liver or to other organs.

Western blot analysis of total cellular 67LR was performed on lysates from cultured cells using two antibodies against 67LR. One of these (HK-149) was raised against a 17-mer synthetic peptide from the N-terminal region of 67LR and the other (HK-48) was raised against a bacterial fusion protein coded for the β-galactosidase gene plus 0.9-kb cDNA sequence from C-terminal region (Clement et al, 1990). Using either antibody, the major protein band migrated at 45 kDa. The mobility and amount of this protein was not different in the three cell lines (Figure 2). Other cross-reactive bands are observed but their amounts are low compared with the 45-kDa band.

Northern blot analysis of cultured cells using 67LR cDNA (Segui-Real et al, 1989) revealed a 1.1-kb transcript. There was no difference in the amount of mRNA in the three cell lines (Figure 3). We conclude that the transcription and expression of 67LR were not different among the three cell lines.

Immunohistochemical localization of 67LR was performed with the HK-149 antibody. In $ET^+$ cells, the antigen was distributed along the cell membrane and in the central lumen-like structure in a linear pattern (Figure 4B). In contrast, $ET^-$ cells did not show a membrane-staining pattern. The 67LR was localized in the cytoplasm of $ET^-$ cells in a spotty distribution (Figure 4C). Parental cells showed diffuse staining both in the membrane and cytoplasm (Figure 4A). The staining pattern was similar, when HK-48 antibody was used instead of HK-149 (data not shown).

Laminin affinity chromatography of cell lysates using a laminin–Sepharose affinity column was carried out. The EDTA-eluted fraction, separated on 7.5% SDS–PAGE under reducing conditions, revealed that the 45-kDa 67LR is more abundant in $ET^+$ cells than in $ET^-$ cells (Figure 5). Lesser amounts of other species that react with antibody are observed and may either be breakdown products (the lower bands) or related species (upper bands) as observed previously (Clement et al, 1990; Kleinman et al, 1991). The same fractions reacted with an antibody to another laminin receptor of 110 kDa. The level of 110-kDa laminin receptor did not differ between the two subclones. These findings suggested that the 67LR is able to bind laminin better on the $ET^+$ cells than on $ET^-$ cells, although the total amounts of 67LR were similar in the two cell lines. Assuming that the binding affinity is higher in the membranous fraction than the cytoplasmic fraction, the above findings corresponds with the different cellular localization of those cell lines. Other explanations are also possible.

### DISCUSSION

We have examined the expression of the 67LR from subclones of colon cancer cell lines that originated from a single patient. Cells from the parental cell line were selected for laminin adhesiveness. We found that laminin-adherent cells had greater tumorigenic ability in orthotopic as well as in heterotopic sites than the non-adherent or parental cells. We also found that the laminin adhesion-selected subclone did not differ in transcription or protein expression of 67LR, whereas the cellular distribution was markedly different. This finding is similar to the differential expression of $\beta_1$ integrin in these subclones, which we demonstrated previously (Kim et al, 1995). We are not sure why both of these adhesion receptors are not fully expressed on the cell surface. A possible defect in protein transport may be involved.

It is not certain why we detect mainly 45-kDa protein in the colon cancer cells. As shown in the Northern blot, the mRNA is 1.1 kb and the deduced amino acid sequence codes for a 32-kDa protein. It is unlikely that the 32-kDa protein forms a dimer to become 67 kDa because reducing agents do not change its molecular weight. It has been proposed that the 67-kDa molecule is a chimeric molecule resulting from post-transcriptional association between the 37-kDa laminin receptor precursor protein (derived from mRNA of 32-kDa amino acid sequence) and a β-galactoside-binding lectin (Castronovo, 1993). Thus, the molecular weight of this protein might vary according to the modification in a cell type-specific manner. Variable-sized proteins (37, 45 or 67 kDa) have been identified with these antibodies (Clement et al, 1990; Weeks et al, 1991).

There is still controversy as to whether 67LR is a surface membrane receptor. Although several researchers have demonstrated that 67LR is a membrane-associated protein that interacts with the cytoskeleton (Cody and Wicha, 1986), Wewer et al (1986) raised the question because the full-length cDNA encoded a much

© Cancer Research Campaign 1998

smaller protein (32 kDa) that lacked a membrane-spanning domain. Our data show that 67LR can be expressed either on the cell membrane or in the cytoplasm, and suggested that the expression pattern might be related to malignancy.

The YIGSR peptide, derived from the laminin $\beta 1$ chain, inhibits tumour growth and metastasis (Iwamoto et al, 1987). This peptide competitively binds to the 67LR (Graf et al, 1987), and it was subsequently demonstrated that YIGSR induced spreading and stress fibre formation through 67LR. The 67LR was co-localized with $\alpha$-actinin and vinculin, which are structural proteins of the cells (Massia et al, 1993). The above findings suggested that the YIGSR is a ligand site of 67LR and that the YIGSR mediated anti-metastatic effect might be due to the competitive blocking the 67LR by soluble peptides.

A strong correlation has been observed between 67LR and human colon carcinomas. Horan-Hand et al (1985) first described an elevated expression of 67LR in colorectal carcinoma compared with normal colonic mucosa or colonic adenoma. Yow et al (1988) isolated the 67LR by differential hybridization using colon cancer cells and normal tissue. The investigation of surgically resected samples and metastatic lesions by Mafune et al (1990) confirmed the increased abundance of 67LR in colon carcinoma, postulating a role of this receptor as a marker of human colorectal cancer progression and aggressiveness. The observation of a higher level of laminin receptor expression on metastatic colon cancer than in the primary tumour (Cioce et al, 1991), strongly suggests that an increased level of laminin receptor expression is associated with a more invasive phenotype and a higher metastatic potential. A positive association of 67LR expression and tumour progression in human gastric carcinoma was also recently demonstrated (Lee et al, 1996). We tried growing these cells orthotopically to see if these cells could metastasize, but no metastatic lesion was observed. This may not be unexpected as the level of 67LR did not change.

Several studies have shown that not only in gastrointestinal cancer but also in human breast cancer, there is increased expression of 67LR, and the level of this receptor appeared to be related to increased invasiveness and metastatic potential (Martignone et al, 1993). Furthermore, oestrogen, which is known to modulate the progression of breast cancer, enhanced the expression of 67LR at both the protein and mRNA level in oestrogen receptor-positive human breast cancer cells (Castronovo et al, 1989). These observations are consistent with the fact that oestrogen receptor-positive breast carcinomas are clinically more aggressive than oestrogen receptor-negative carcinomas. Oestrogen-induced exacerbation of oestrogen receptor-positive breast cancer could also be related to the up-regulation of 67LR.

In summary, this study revealed that subcloned colon cancer cells of varying malignant potential exhibited a different expression pattern of 67LR. Based on our previous work with these cells (Kim et al, 1995), we find that the adhesion selection altered the distribution of at least two potential laminin receptors, including $\beta_1$ integrin and 67LR. These alterations could be responsible for the difference in laminin adhesiveness, invasiveness and tumorigenicity.

### ACKNOWLEDGEMENTS

This work was supported by a research grant from the Sam Mi Cultural Foundation (1995) on behalf of the Korean Medical Association, and the Non Directed Research Fund, Korea Research Foundation (01-F-0079, 1995).

### REFERENCES

Aznavoorian S, Liotta LA and Kupchik HK (1990) Characteristics of invasive and non-invasive human colorectal adenocarcinoma cells. *J Natl Cancer Inst* **80**: 1485–1492

Castronovo V (1993) Laminin receptors and laminin-binding proteins during tumor invasion and metastasis. *Inv Metastas* **3**: 1–30

Castronovo V, Taraboletti G, Liotta LA and Sobel ME (1989) Modulation of laminin receptor expression by estrogen and progestins in human breast cancer cell lines. *J Natl Cancer Inst* **81**: 781–788

Cioce V, Castronovo V, Shmookler BM, Garbisa S, Grigioni WF, Liotta LA and Sobel ME (1991) Increased expression of the laminin receptors of human colon cancer. *J Natl Cancer Inst* **83**: 29–36

Clement B, Bartolome SR, Pierre S, Kleinman HK and Yamada Y (1990) Hepatocyte attachment to laminin is mediated through multiple receptors. *J Cell Biol* **110**: 185–192

Cody RL and Wicha MS (1986) Clustering of cell surface laminin enhances its association with the cytoskeleton. *Exp Cell Res* **165**: 107–116

Graf J, Yamada Y, Robey FA, Sasaki M, Ogle RC, Iwamoto Y, Kleinman HK and Martin GR (1987) A pentapeptide from the B1 chain of laminin promotes cell attachment and bind to the 67 kD laminin receptor. *Biochemistry* **26**: 6896–6900

Horan-Hand P, Thor A, Schlom J, Rao, CN and Liotta LA (1985) Expression of laminin receptor in normal and carcinomatous tissue as defined by monoclonal antibody. *Cancer Res* **45**: 2713–2719

Iwamoto Y, Robey FA, Graf J, Sasaki M, Kleinman HK, Yamada Y and Martin GR (1987) YIGSR a pentapeptide from the B1 chain of laminin inhibits tumor cell metastasis. *Science* **238**: 1132–1134

Jun SH, Thormpson EW, Gottardis M, Torri J, Yamamura K, Kibbey MC, Kim WH and Kleinman HK (1994) Laminin adhesion-selected primary human colon cancer cells are more tumorigenic than the parental and non adherent cells. *Int J Oncol* **4**: 55–60

Kim WH, Schnaper HW, Nomizu M, Yamada Y and Kleinman HK (1994) Apoptosis in human fibrosarcoma cells is induced by a multimeric synthetic Tyr-Ile-Gly-Ser-Arg (YIGSR)-containing polypeptide from laminin. *Cancer Res* **54**: 5005–5010

Kim WH, Jun SH, Kibbey MC, Thompson EW and Kleinman HK (1995) Expression of $\beta_1$ integrin in laminin-adhesion-selected human colon cancer cell lines of varying tumorigenicity. *Inv Metastas* **14**: 147–155

Kleinman HK, Mcgarvey ML, Hassell JR, Star VL, Cannon FB, Laurie GW and Martin GR (1986) Basement membrane complexes with biological activity. *Biochemistry* **25**: 312–318

Kleinman HK, Weeks BS, Cannon FB, Sweeney TM, Sephel GC, Clement B, Zain M, Olson MO, Jucker M and Burrous BA (1991) Identification of a 110-kDa nonintegrin cell surface laminin-binding protein which recognizes an A chain neurite-promoting peptide. *Arch Biochem Biophys* **290**: 320–325

Kleinman HK, Weeks BS, Schnaper WH, Kibbey MC, Yamamura K and Grant DS (1993) The laminins: A family of basement membrane glycoproteins important in cell differentiation and tumor metastases. *Vitam Horm* **47**: 161–186

Lee WA, Kim WH, Kim YI, Kim JP and Kleinman HK (1996) Overexpression of 67 kD laminin receptor correlates with the progression of gastric carcinoma. *Path Res Pract* **192**: 1195–1201

Mafune K, Ravikumar TS, Wong JM, Yow H, Chen LB and Steele GD (1990) Expression of a Mr 32,000 laminin-binding protein messenger RNA in human colon carcinoma correlates with disease progression. *Cancer Res* **50**: 3888–3891

Malinoff HL and Wicha MS (1983) Isolation of a cell surface receptor protein for laminin from murine fibrosarcoma cells. *J Cell Biol* **96**: 1475–1479

Martignone S, Menard S, Bufalino R, Cascinelli N, Pellegrine R, Tagliabue E, Andreola S, Rilke F and Colnaghi MI (1993) Prognostic significance of the 67-kilodalton laminin receptor expression in human breast carcinomas. *J Natl Cancer Inst* **85**: 398–402

Martin GR and Timpl RL (1987) Laminin and other basement membrane components. *Annu Rev Cell Biol* **3**: 57–85

Massia SP, Rao SS and Hubbell JA (1993) Covalently immobilized laminin peptide Tyr-Ile-Gly-Ser-Arg (YIGSR) supports cell spreading and co-localization of the 67 kD laminin receptor with $\alpha$-actinin and vinculin. *J Biol Chem* **268**: 8053–8059

Segui-Real B, Rhodes C and Yamada Y (1989) The human genome contains a pseudogene for the Mr=32,000 laminin binding protein. *Nucleic Acids Res* **17**: 1257

Terranova VP, Wiliam JE, Liotta LA and Martin GR (1984) Modulation of the metastatic activity of melanoma cells by laminin and fibronectin. *Science* **226**: 982–985

Togo S, Shimada H, Kubota T, Moossa AR and Hoffman RM (1995) Host organ specifically determines cancer progression. *Cancer Res* **55**: 681–684

© Cancer Research Campaign 1998

Weeks BS, Kopp JB, Horikoshi S, Cannon FB, Garrett M, Kleinman HK and Klotman PE (1991) Adult and fetal human mesangial cells interact with specific laminin domains. *Am J Physiol* **30**: F688–F695

Wewer UM, Liotta LA, Jaye M, Ricca GA, Drohan WN, Claysmith AP, Rao CN, Wirth P, Coligan JE, Albrechtsen R, Mudryi M and Sobel ME (1986) Altered levels of laminin receptor mRNA in various human carcinoma cells that have different abilities to bind laminin. *Proc Natl Acad Sci USA* **83**: 7137–7141

Yamamura K, Kibbey MC and Kleinman HK (1993) Melanoma cells selected for adhesion to laminin peptides have different malignant properties. *Cancer Res* **53**: 423–428

Yow H, Weng JM, Chen HS, Lee C, Steele GDJ and Chen LB (1988) Increased mRNA expression of a laminin-binding protein in human colon carcinoma: Complete sequence of a full-length cDNA encoding the protein. *Proc Natl Acad Sci USA* **81**: 1974–1980

© Cancer Research Campaign 1998


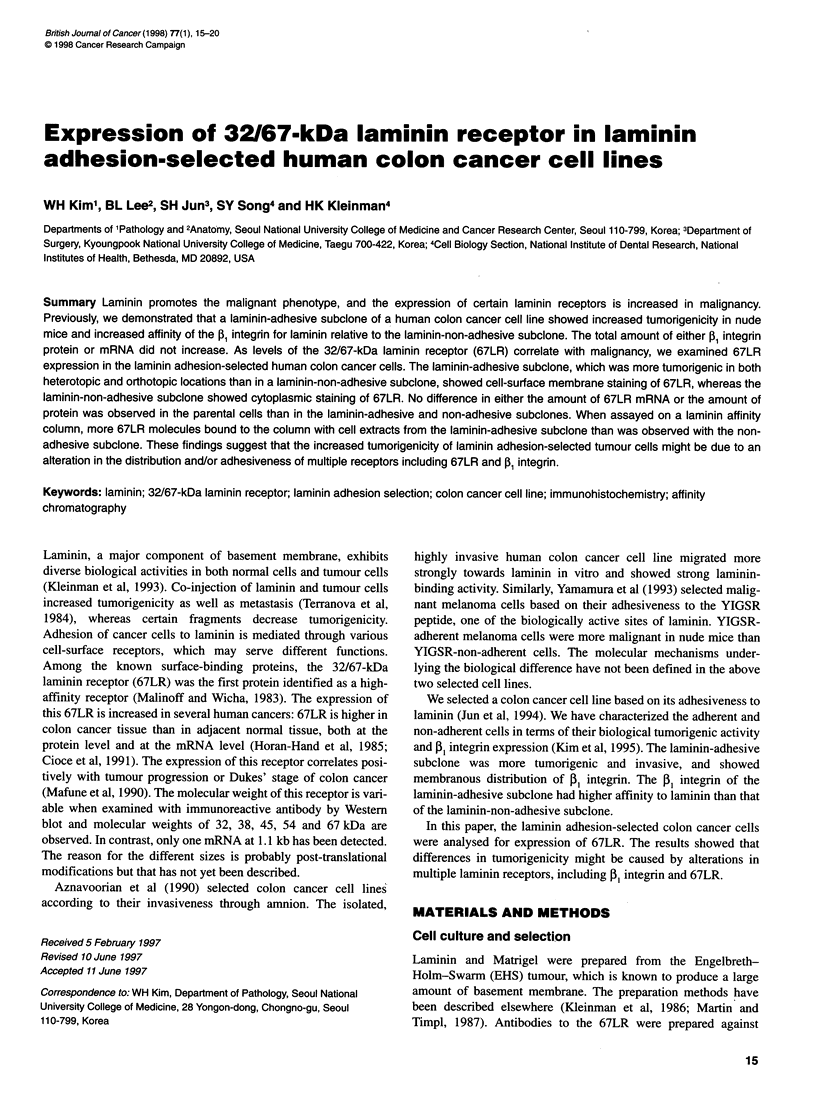

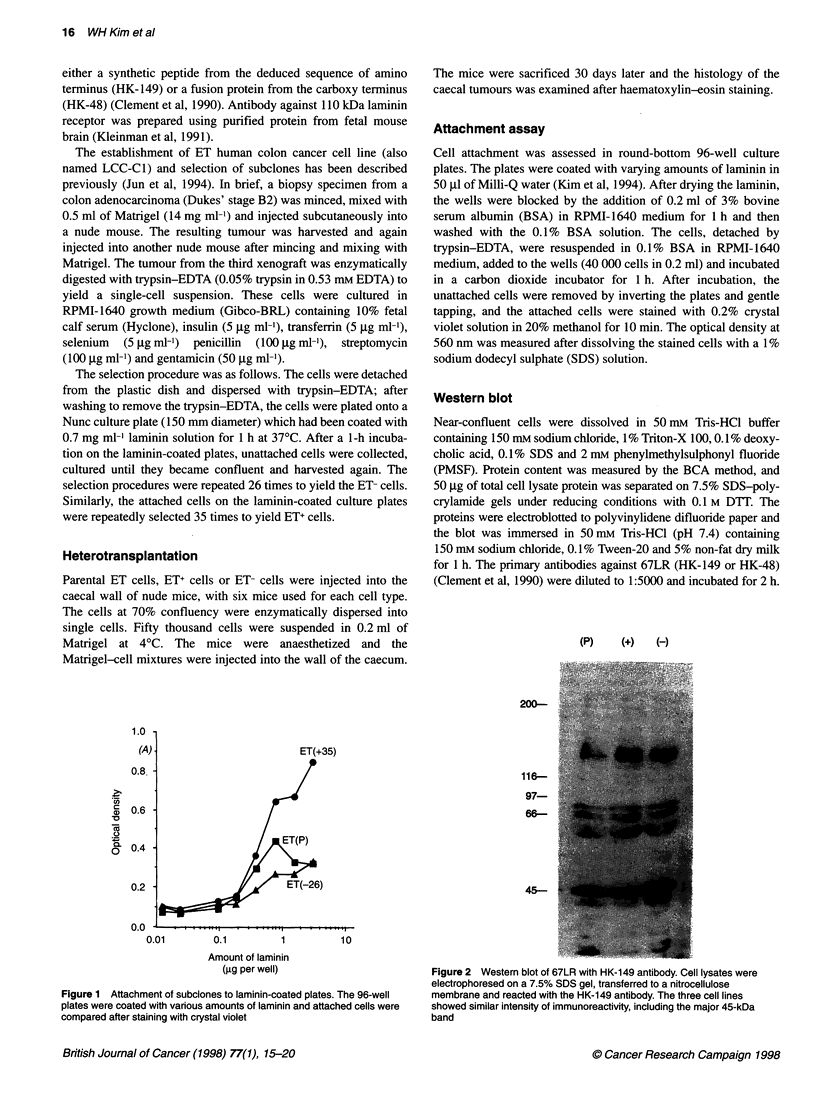

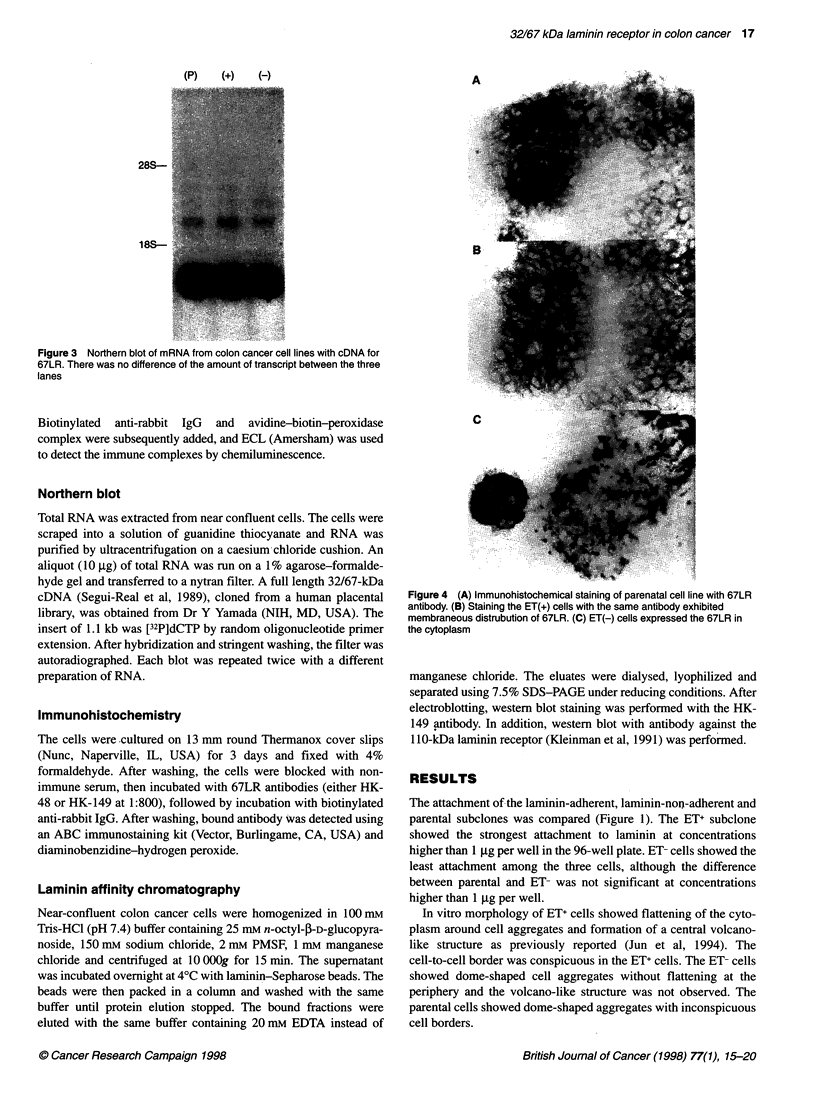

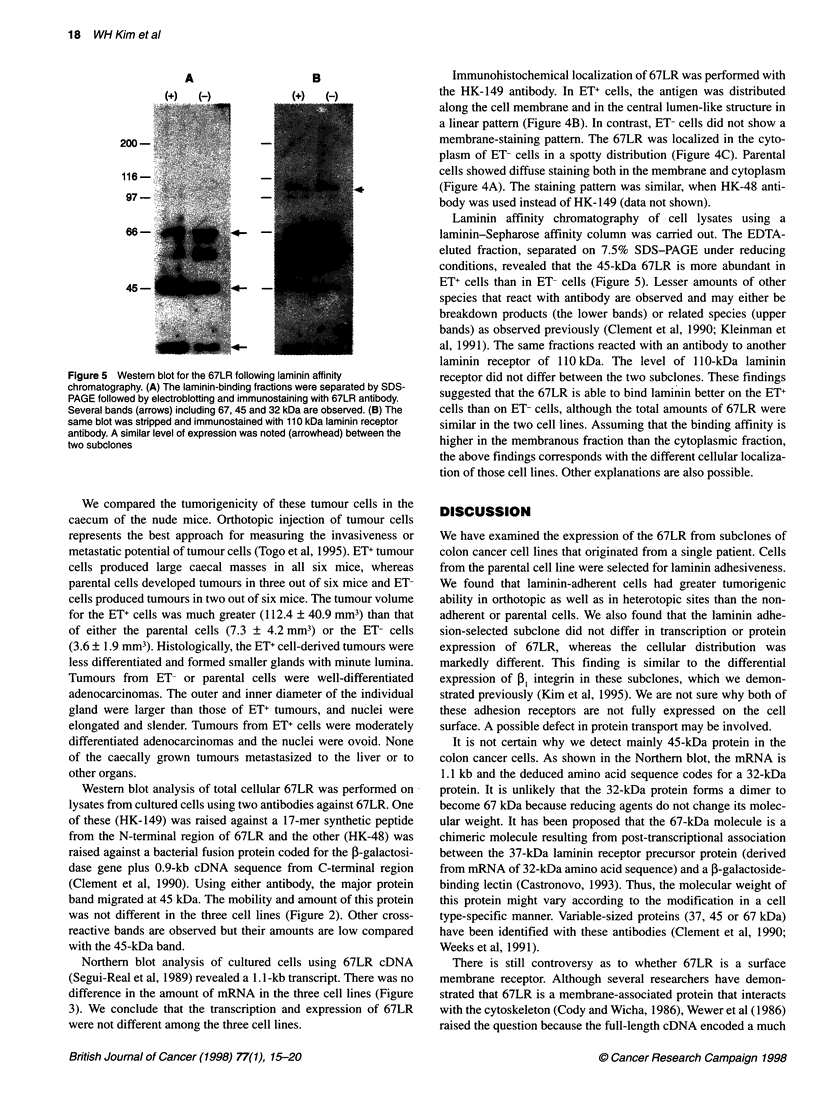

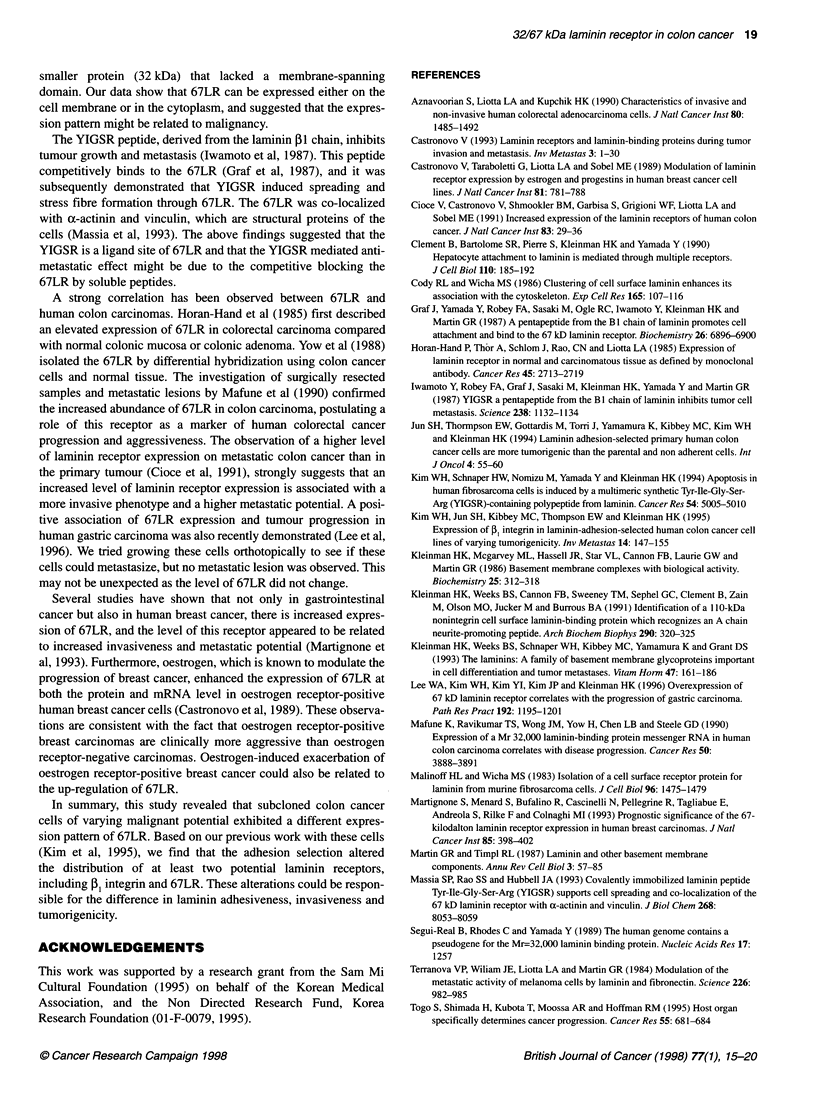

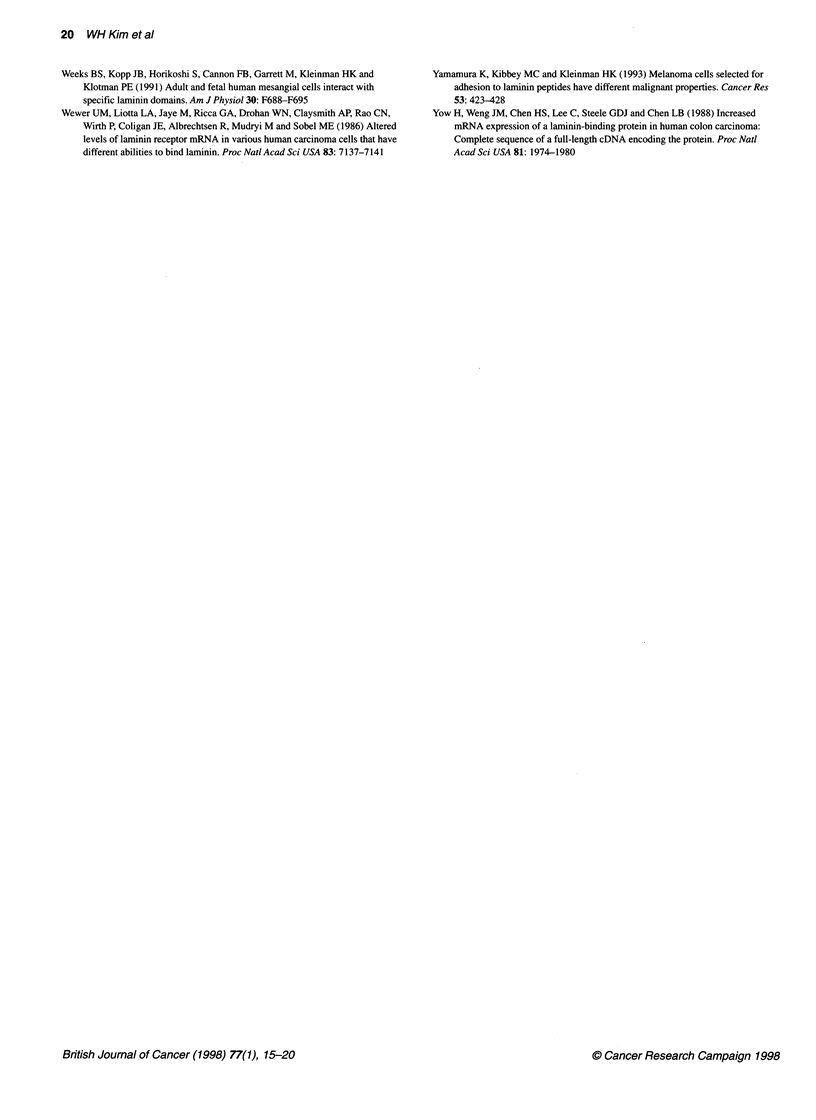


## References

[OCR_00515] Aznavoorian S., Liotta L. A., Kupchik H. Z. (1990). Characteristics of invasive and noninvasive human colorectal adenocarcinoma cells.. J Natl Cancer Inst.

[OCR_00520] Castronovo V. (1993). Laminin receptors and laminin-binding proteins during tumor invasion and metastasis.. Invasion Metastasis.

[OCR_00524] Castronovo V., Taraboletti G., Liotta L. A., Sobel M. E. (1989). Modulation of laminin receptor expression by estrogen and progestins in human breast cancer cell lines.. J Natl Cancer Inst.

[OCR_00529] Cioce V., Castronovo V., Shmookler B. M., Garbisa S., Grigioni W. F., Liotta L. A., Sobel M. E. (1991). Increased expression of the laminin receptor in human colon cancer.. J Natl Cancer Inst.

[OCR_00534] Clément B., Segui-Real B., Savagner P., Kleinman H. K., Yamada Y. (1990). Hepatocyte attachment to laminin is mediated through multiple receptors.. J Cell Biol.

[OCR_00539] Cody R. L., Wicha M. S. (1986). Clustering of cell surface laminin enhances its association with the cytoskeleton.. Exp Cell Res.

[OCR_00543] Graf J., Ogle R. C., Robey F. A., Sasaki M., Martin G. R., Yamada Y., Kleinman H. K. (1987). A pentapeptide from the laminin B1 chain mediates cell adhesion and binds the 67,000 laminin receptor.. Biochemistry.

[OCR_00548] Hand P. H., Thor A., Schlom J., Rao C. N., Liotta L. (1985). Expression of laminin receptor in normal and carcinomatous human tissues as defined by a monoclonal antibody.. Cancer Res.

[OCR_00553] Iwamoto Y., Robey F. A., Graf J., Sasaki M., Kleinman H. K., Yamada Y., Martin G. R. (1987). YIGSR, a synthetic laminin pentapeptide, inhibits experimental metastasis formation.. Science.

[OCR_00569] Kim W. H., Jun S. H., Kibbey M. C., Thompson E. W., Kleinman H. K. (1994). Expression of beta 1 integrin in laminin-adhesion-selected human colon cancer cell lines of varying tumorigenicity.. Invasion Metastasis.

[OCR_00565] Kim W. H., Schnaper H. W., Nomizu M., Yamada Y., Kleinman H. K. (1994). Apoptosis in human fibrosarcoma cells is induced by a multimeric synthetic Tyr-Ile-Gly-Ser-Arg (YIGSR)-containing polypeptide from laminin.. Cancer Res.

[OCR_00574] Kleinman H. K., McGarvey M. L., Hassell J. R., Star V. L., Cannon F. B., Laurie G. W., Martin G. R. (1986). Basement membrane complexes with biological activity.. Biochemistry.

[OCR_00579] Kleinman H. K., Weeks B. S., Cannon F. B., Sweeney T. M., Sephel G. C., Clement B., Zain M., Olson M. O., Jucker M., Burrous B. A. (1991). Identification of a 110-kDa nonintegrin cell surface laminin-binding protein which recognizes an A chain neurite-promoting peptide.. Arch Biochem Biophys.

[OCR_00585] Kleinman H. K., Weeks B. S., Schnaper H. W., Kibbey M. C., Yamamura K., Grant D. S. (1993). The laminins: a family of basement membrane glycoproteins important in cell differentiation and tumor metastases.. Vitam Horm.

[OCR_00590] Lee W. A., Kim W. H., Kim Y. I., Yang H. K., Kim J. P., Kleinman H. K. (1996). Overexpression of the 67 kD laminin receptor correlates with the progression of gastric carcinoma.. Pathol Res Pract.

[OCR_00595] Mafune K., Ravikumar T. S., Wong J. M., Yow H., Chen L. B., Steele G. D. (1990). Expression of a Mr 32,000 laminin-binding protein messenger RNA in human colon carcinoma correlates with disease progression.. Cancer Res.

[OCR_00601] Malinoff H. L., Wicha M. S. (1983). Isolation of a cell surface receptor protein for laminin from murine fibrosarcoma cells.. J Cell Biol.

[OCR_00605] Martignone S., Ménard S., Bufalino R., Cascinelli N., Pellegrini R., Tagliabue E., Andreola S., Rilke F., Colnaghi M. I. (1993). Prognostic significance of the 67-kilodalton laminin receptor expression in human breast carcinomas.. J Natl Cancer Inst.

[OCR_00611] Martin G. R., Timpl R. (1987). Laminin and other basement membrane components.. Annu Rev Cell Biol.

[OCR_00615] Massia S. P., Rao S. S., Hubbell J. A. (1993). Covalently immobilized laminin peptide Tyr-Ile-Gly-Ser-Arg (YIGSR) supports cell spreading and co-localization of the 67-kilodalton laminin receptor with alpha-actinin and vinculin.. J Biol Chem.

[OCR_00621] Segui-Real B., Rhodes C., Yamada Y. (1989). The human genome contains a pseudogene for the Mr=32,000 laminin binding protein.. Nucleic Acids Res.

[OCR_00626] Terranova V. P., Williams J. E., Liotta L. A., Martin G. R. (1984). Modulation of the metastatic activity of melanoma cells by laminin and fibronectin.. Science.

[OCR_00631] Togo S., Shimada H., Kubota T., Moossa A. R., Hoffman R. M. (1995). Host organ specifically determines cancer progression.. Cancer Res.

[OCR_00639] Weeks B. S., Kopp J. B., Horikoshi S., Cannon F. B., Garrett M., Kleinman H. K., Klotman P. E. (1991). Adult and fetal human mesangial cells interact with specific laminin domains.. Am J Physiol.

[OCR_00644] Wewer U. M., Liotta L. A., Jaye M., Ricca G. A., Drohan W. N., Claysmith A. P., Rao C. N., Wirth P., Coligan J. E., Albrechtsen R. (1986). Altered levels of laminin receptor mRNA in various human carcinoma cells that have different abilities to bind laminin.. Proc Natl Acad Sci U S A.

[OCR_00650] Yamamura K., Kibbey M. C., Kleinman H. K. (1993). Melanoma cells selected for adhesion to laminin peptides have different malignant properties.. Cancer Res.

