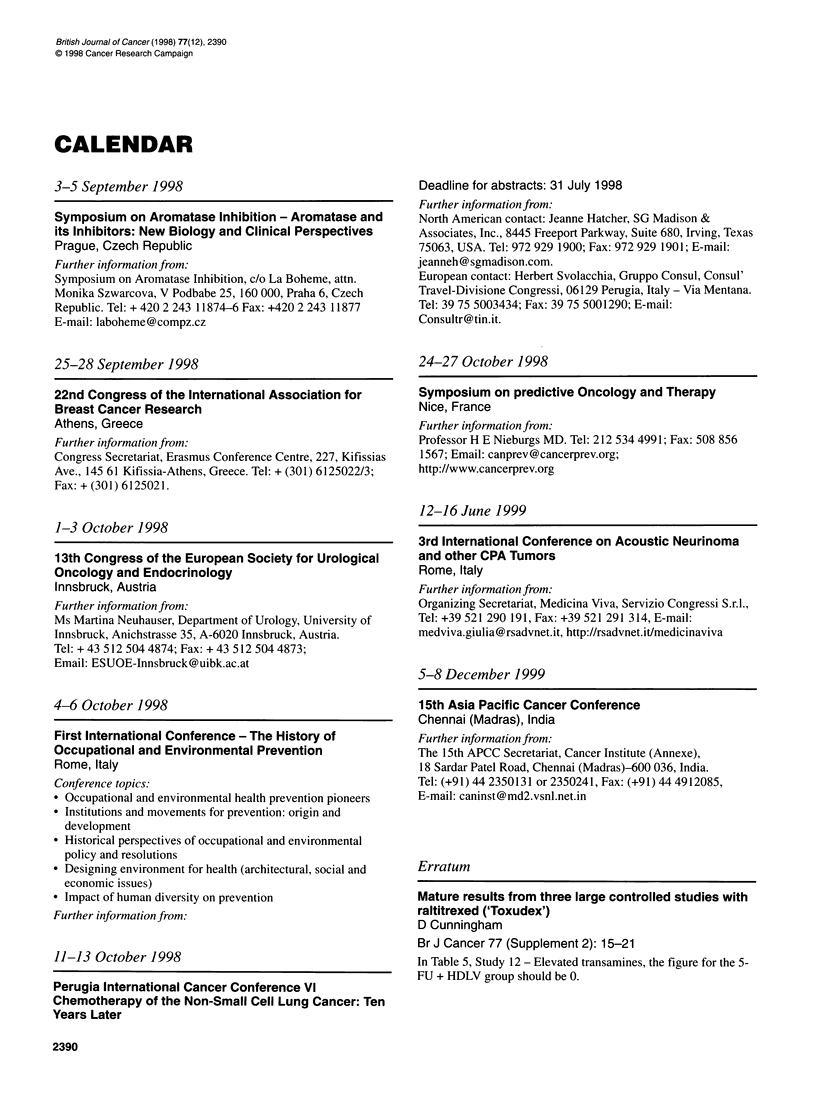# Mature results from three large controlled studies with raltitrexed ('Toxudex')

**Published:** 1998-06

**Authors:** 


*Erratum*

**Mature results from three large controlled studies with raltitrexed ('Toxudex')**
D Cunningham

Br J Cancer 77 (Supplement 2): 15–21

In Table 5, Study 12 – Elevated transamines, the figure for the 5-FU + HDLV group should be 0.